# Caloric restriction prevents obesity- and intermittent hypoxia-induced cardiac remodeling in leptin-deficient *ob/ob* mice

**DOI:** 10.3389/fphys.2022.963762

**Published:** 2022-09-08

**Authors:** Aaron A. Jones, Sarah N. Framnes-DeBoer, Arianne Shipp, Deanna M. Arble

**Affiliations:** ^1^ Department of Biological Sciences, Marquette University, Milwaukee, WI, United States; ^2^ Kiran C. Patel College of Osteopathic Medicine, Nova Southeastern University, Clearwater, FL, United States

**Keywords:** sleep apnea, leptin, obesity, intermittent hypoxia (IH), caloric restriction, echocardiography, left ventricle (LV), cardiac remodeling

## Abstract

**Background:** Intermittent hypoxia (IH), a key characteristic of obstructive sleep apnea, is independently associated with cardiometabolic impairment. While endogenous leptin levels may provide cardioprotective effects against hypoxia, leptin resistance is common among obese individuals presenting with obstructive sleep apnea.

**Methods:** Here, we assessed left ventricle (LV) function using M-mode echocardiography in lean wild-type, calorically-restricted *ob/ob*, and obese *ob/ob* mice before and after 6 days of IH to determine how obesity and intermittent hypoxia interact to affect cardiac function independent of leptin signaling.

**Results:** Calorically-restricting *ob/ob* mice for 4 weeks prior to IH exposure prevented weight gain (−2.1 ± 1.4 g) compared to free-fed *ob/ob* mice (8.7 ± 1.1 g). Free-fed *ob/ob* mice exhibited increased LV mass (0.713 ± 0.008 g) relative to wild-type mice (0.685 ± 0.004 g) and increased posterior wall thickness (0.089 ± 0.006 cm) relative to calorically-restricted *ob/ob* mice (0.072 ± 0.004 cm). Following 6 days of IH, free-fed *ob/ob* mice exhibited increases in cardiac output (44.81 ± 2.97 pre-IH vs. 57.14 ± 3.09 ml/min post-IH), LV diameter (0.400 ± 0.007 pre-IH vs. 0.428 ± 0.009 cm post-IH) and end diastolic volume (0.160 ± 0.007 pre-IH vs. 0.195 ± 0.012 ml post-IH) that were not detected in wild-type or calorically-restricted *ob/ob* mice.

**Conclusion:** Caloric restriction can prevent obesity-induced LV hypertrophy and protect against acute IH-induced cardiac remodeling independent of leptin signaling. These findings may have clinical implications for obstructive sleep apnea.

## Introduction

Obstructive sleep apnea (OSA) is a prevalent sleep disorder characterized by periodic cessations of breathing during sleep. Intermittent hypoxia (IH) is a key characteristic of OSA and has detrimental consequences on cardiometabolic health (Polotsky et al., 2003). Mice exposed to IH, even for acute periods, develop cardiometabolic impairments including increased ventricular pressure, hypertension, and hyperlipidemia (Campen et al., 2005; Li et al., 2005). Moreover, chronic IH can cause cardiac remodeling and changes in left ventricle (LV) contractility in mice (Naghshin et al., 2009; Naghshin et al., 2012), which is suspected of contributing to the development of heart failure in individuals with OSA (Sanderson et al., 2021).

IH increases circulating levels of the hormone leptin in both rodents and humans (Grosfeld et al., 2002; Messenger and Ciriello, 2013). Moreover, individuals with OSA have higher circulating leptin levels independent of body fat (Ip et al., 2000). Elevated leptin levels, which often coincide with leptin resistance (Framnes and Arble, 2018), are correlated with hypertension, myocardial infarction, and stroke (Koh et al., 2008). Leptin treatment reduces cardiac wall thickness and cardiomyocyte hypertrophy in leptin-deficient *ob/ob* mice independent of obesity (Barouch et al., 2003), and protects cardiomyocytes from hypoxia-induced apoptosis (Shin et al., 2009), suggesting leptin exhibits cardioprotective effects against obesity and hypoxia. It is unclear whether leptin resistance or other aspects of obesity make individuals with OSA more susceptible to IH-induced cardiac remodeling, warranting further investigation.

Caloric restriction has been shown to reduce LV hypertrophy and improve diastolic function in obese individuals (Iacobellis et al., 2008) and obese rodent models (Karwi et al., 2019; An et al., 2020). The cardioprotective effects of caloric restriction can occur independent of weight loss (Ahmet et al., 2011) and leptin signaling (Xu et al., 2016; An et al., 2020). However, it remains unknown whether caloric restriction can also mitigate IH-induced cardiac remodeling, as no studies have investigated the effects of caloric restriction on cardiac function in rodents exposed to IH or humans with OSA.

In the present study, we exposed calorically-restricted, leptin-deficient *ob/ob* mice to 6 days of IH to investigate whether acute IH exacerbates LV dysfunction in an obese model of leptin deficiency and whether caloric restriction can mitigate these effects. Overall, we found that obese, leptin-deficient *ob/ob* mice developed LV hypertrophy and were more susceptible to IH-induced cardiac remodeling. However, these changes in cardiac function were not observed following IH in either wild-type or caloric-restricted *ob/ob* mice. Taken together, caloric restriction prevented obesity-induced LV hypertrophy and protected against acute IH-induced cardiac remodeling independent of leptin signaling in obese mice.

## Materials and methods

### Animals

Eight-week-old male C57BL/6J wild-type (WT) mice and leptin-deficient *ob/ob* mice were ordered from the Jackson Laboratory (Bar Harbor, ME, United States) and fed a standard diet (Teklad Diet 8604) under 12L:12D light conditions. WT (*n* = 6) and a subset of *ob/ob* mice (*n* = 8) were fed *ad-libitum.* For 4 weeks, a subset of *ob/ob* mice (*n* = 6) were calorically-restricted to match the daily food intake of WT mice. Weighed food was provided to calorically-restricted *ob/ob* mice daily between 10 a.m. and 5 p.m. All mice were single-housed. ​Body weight and food intake were measured daily.

### Intermittent hypoxia

At 12 weeks old, all mice were exposed to intermittent hypoxia (IH) over the course of 6 days. IH was delivered for 9 h each day from 9 a.m. to 6 p.m. Mice were kept in their home cages and placed into an IH chamber (BioSpherix Quick & Quiet System, Parish, NY, United States) which cycled 30-s bouts of 5% O_2_ desaturation occurring once every 6 min (10 cycles/hour), as previously described (Framnes-DeBoer et al., 2021).

### Cardiac function

Transthoracic M-mode echocardiography (Syed et al., 2005) was performed at the Medical College of Wisconsin (Milwaukee, WI, United States) by a trained technician using an 11 MHz M12L linear array transducer interfaced to a Vivid 7 General Electric ultrasound machine. All mice were anesthetized during the procedure. Isoflurane (1.5%–2.5%) was delivered to be the least necessary to anesthetize the animal, as measured by failure to respond to a toe pinch. Once an adequate plane of anesthesia was achieved, mice remained under anesthesia for approximately 5 min during which all measurements were collected. Mice remained on a water-circulating heating pad for the duration of anesthesia. Body temperature was not monitored. LV measurements including heart rate, end diastolic and systolic volumes, mass, posterior wall and interventricular septum thickness, and internal diameter during diastole and systole were taken. All variables measured in centimeters were measured with a resolution of 0.01 cm. Heart rate was averaged over three cardiac cycle lengths from M-mode images. End diastolic and systolic volumes were calculated using the Teichholz formula: 7D^3^/(2.4 + D) (Teichholz et al., 1976). LV mass was calculated using 1.05*[(LVEDD^3^ + IVS^3^ + PW^3^)−(LVEDD^3^)] (Syed et al., 2005). Stroke volume was calculated by subtracting end systolic volume from end diastolic volume. Cardiac output was calculated by multiplying stroke volume by heart rate. Ejection fraction was calculated using (SV/EDV)*100. Fractional shortening was calculated using (LVEDD−LVESD/LVEDD)*100. Mice received an echocardiogram after 4 weeks of caloric restriction but before IH, and again immediately following 6 days of IH.

### Statistical analysis

All statistics were performed using GraphPad Prism 9. All cardiac data and Supplementary Figures 1A,B were analyzed by 2-way ANOVA with Tukey’s or Sidak’s multiple comparisons tests. Supplementary Figures 1C,D were analyzed using one-sample t-tests. Values are reported as the mean ± SEM. All graphs report significant differences using a 2-tailed test design where *p < 0.05, **p < 0.01, ***p < 0.001, and ****p < 0.0001. ROUT tests (Q = 1%) were performed to verify no outliers were present in the data.

## Results

### Caloric restriction prevents weight gain in *ob/ob* mice

Prior to 4 weeks of caloric restriction, our two groups of *ob/ob* mice weighed similarly (45.3 ± 1.2 vs. 43.9 ± 1.5 g) and were significantly heavier than WT mice (23.3 ± 0.25 g; Supplementary Figure 1A). Calorically-restricted *ob/ob* mice were fed an average of 4.1 ± 0.1 g/day which was determined by the daily amount consumed by WT mice (4.4 ± 0.2 g/day; Supplementary Figure 1B). This was significantly less than the amount consumed by free-fed *ob/ob* mice who ate an average of 6.2 ± 0.6 g/day over the same period. Caloric restriction prevented weight gain in *ob/ob* mice (*p* = 0.21; Supplementary Figure 1C), resulting in a final weight of 41.8 ± 0.64 g (Supplementary Figure 1A). In contrast, free-fed *ob/ob* mice gained 8.7 ± 1.1 g (*p* < 0.0001; Supplementary Figure 1C) over the 4-week period, resulting in a final weight of 54.1 ± 2.2 g (Supplementary Figure 1A). Despite preventing weight gain, calorically-restricted *ob/ob* mice still weighed substantially more than WT mice (26.7 ± 0.37 g) after the 4-week caloric restriction period (Supplementary Figure 1A). All mice were then exposed to IH for 6 days. During IH, WT and calorically-restricted *ob/ob* mice continued to eat significantly less than free-fed *ob/ob* mice. There was no effect of IH exposure on daily food intake for any group (Supplementary Figure 1B). WT, but not free-fed or calorically-restricted *ob/ob* mice, lost weight during IH (Supplementary Figure 1D).

### Caloric restriction protects against intermittent hypoxia-induced increases in cardiac workload

**FIGURE 1:**
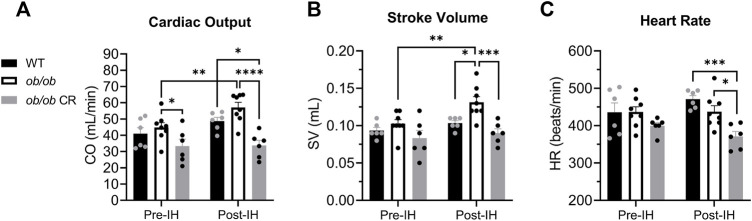
Caloric restriction protects ob/ob mice from IH-induced increases in cardiac workload. **(A)** Cardiac output was lower in calorically-restricted *ob/ob* mice (*n* = 6) before IH relative to free-fed *ob/ob* mice (*n* = 8). The cardiac output of free-fed *ob/ob* mice was increased following IH exposure and remained significantly higher than calorically-restricted *ob/ob* mice. **(B)** Post-IH, stroke volume was increased in free-fed *ob/ob* mice and significantly elevated compared to calorically-restricted *ob/ob* and WT mice (*n* = 6). **(C)** Following IH exposure, calorically-restricted *ob/ob* mice had a lower heart rate than WT and free-fed *ob/ob* mice. Abbreviations: CR, (calorically-restricted).

Prior to IH, heart rate and stroke volume were similar among all groups. However, cardiac output was reduced in calorically-restricted *ob/ob* mice as compared to their free-fed counterparts (Figure 1A). Following IH, free-fed *ob/ob* mice exhibited an increase in cardiac output (Figure 1A) *via* an increased stroke volume (Figure 1B) that was not observed in WT or calorically-restricted *ob/ob* mice. This IH-induced increase resulted in free-fed *ob/ob* mice having a significantly increased cardiac output and stroke volume compared to the calorically-restricted *ob/ob* mice post-IH. Following IH, calorically-restricted *ob/ob* mice exhibited a lower heart rate compared to WT and free-fed *ob/ob* mice (Figure 1C), however, their heart rate was not significantly different from their pre-IH measures. Overall, we found that caloric restriction protects against IH-induced increases in cardiac workload.

### Left ventricle systolic function is preserved in *ob/ob* mice exposed to intermittent hypoxia

**FIGURE 2:**
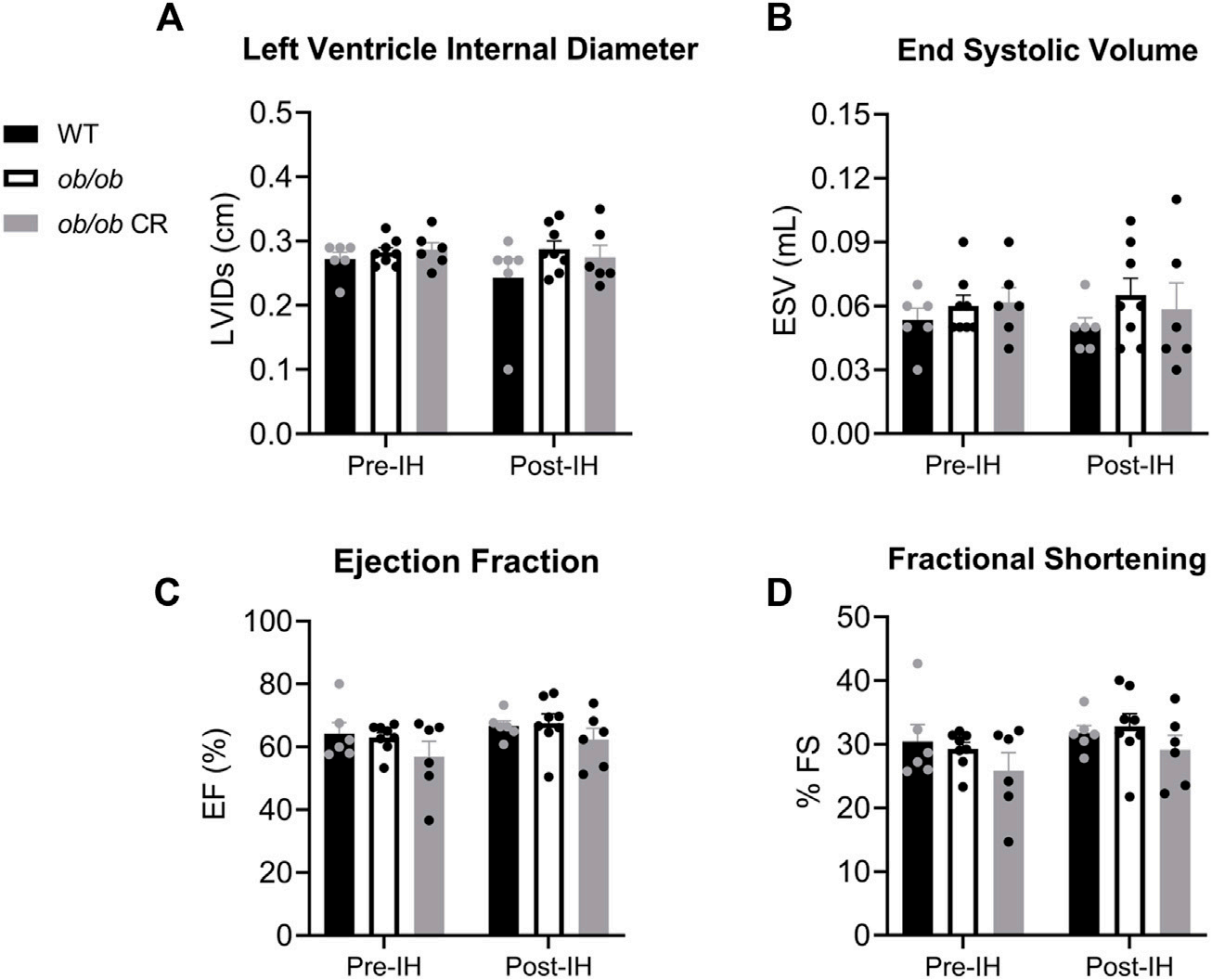
LV systolic function is preserved in *ob/ob* mice and unaffected by 6 days of IH. The **(A)** LV internal diameter during systole and **(B)** end systolic volume were unaltered in free-fed *ob/ob* mice (*n* = 8) relative to WT (*n* = 6) and calorically-restricted *ob/ob* mice (*n* = 6), and unaffected by IH exposure. Both **(C)** ejection fraction and **(D)** fractional shortening were preserved in all groups pre- and post-IH. Abbreviations: CR, (calorically-restricted).

To determine how obesity and IH contribute to cardiac remodeling in *ob/ob* mice, we next examined systolic and diastolic LV function, separately. Overall, LV systolic function was similar among all our groups and was unchanged by IH exposure. WT, free-fed *ob/ob*, and calorically-restricted *ob/ob* mice all exhibited similar LV diameter during systole (Figure 2A) and end systolic volume (Figure 2B) before and after IH. Similarly, the ejection fraction (Figure 2C) and fractional shortening (Figure 2D) of the LV during systole was preserved in free-fed *ob/ob* mice relative to WT and calorically-restricted *ob/ob* mice both before and after IH.

### Left ventricle diastolic function is altered in free-fed but not calorically-restricted *ob/ob* mice exposed to intermittent hypoxia

**FIGURE 3:**
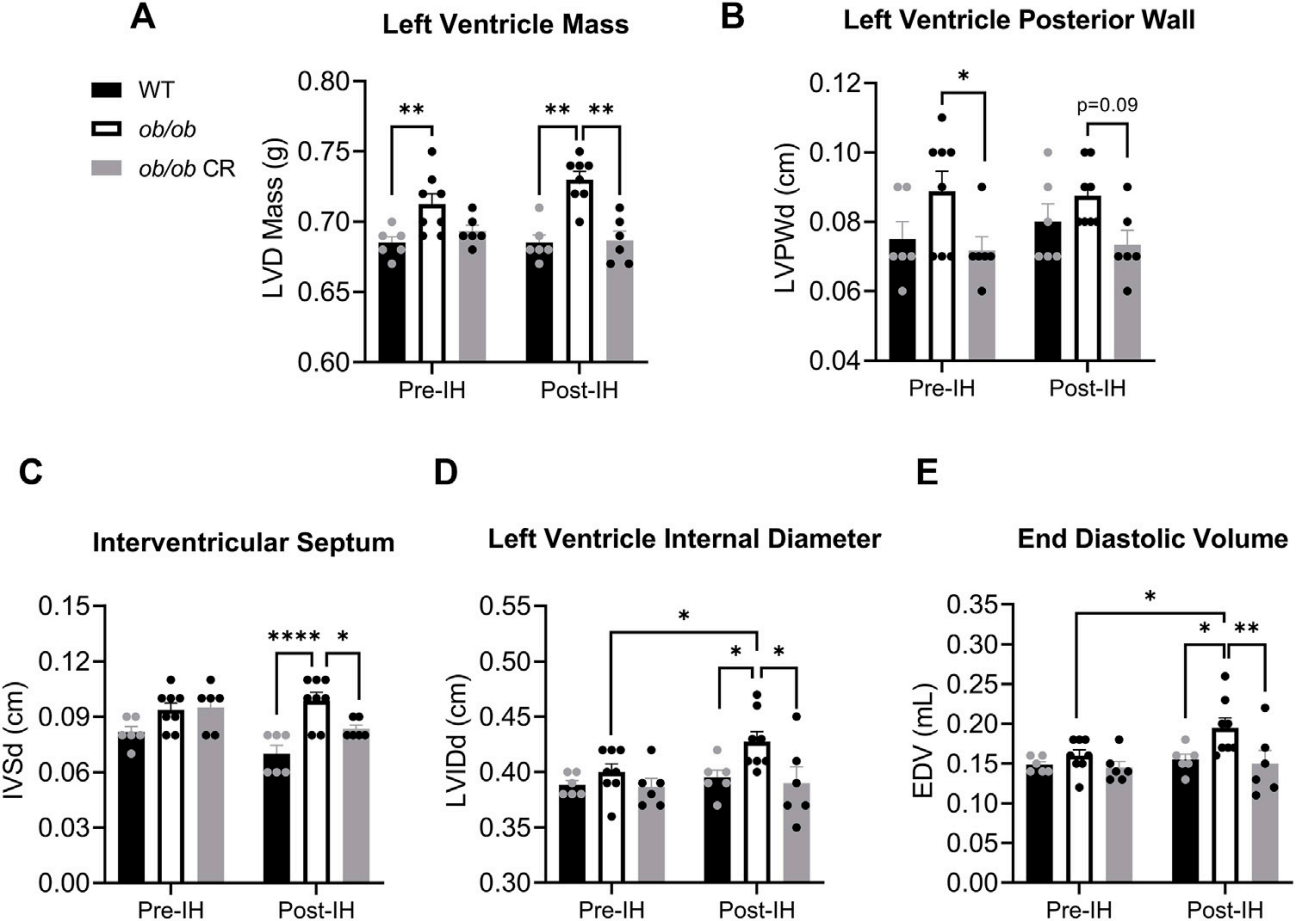
Caloric restriction protects ob/ob mice from LV hypertrophy and IH-induced ventricular dilation. The **(A)** LV mass was higher in free-fed *ob/ob* mice (*n* = 8) relative to WT (*n* = 6) pre-IH, and larger relative to calorically-restricted *ob/ob* (*n* = 6) and WT post-IH. The **(B)** posterior wall thickness within the LV was decreased in calorically-restricted *ob/ob* mice relative to free-fed *ob/ob* pre-IH. The **(C)** interventricular septum thickness was increased in free-fed *ob/ob* relative to WT and calorically-restricted *ob/ob* mice post-IH. The **(D)** LV internal diameter during diastole and **(E)** end diastolic volume increased in free-fed *ob/ob* mice following IH exposure. Abbreviations: CR (calorically-restricted).

Similar to previous findings (Barouch et al., 2003), we found that prior to IH, free-fed *ob/ob* mice exhibited LV hypertrophy as demonstrated by an increased LV mass (Figure 3A) and posterior wall thickness (Figure 3B) relative to WT and calorically-restricted *ob/ob* mice, respectively. LV hypertrophy was mitigated by caloric restriction independent of IH exposure. Following IH, the LV hypertrophy observed in *ob/ob* free-fed mice persisted, with the addition of interventricular septum thickness being significantly higher in free-fed *ob/ob* mice (Figure 3C). However, it is unclear if this was due to an IH-induced increase in septum thickness in free-fed *ob/ob* mice or an IH-induced decrease in the other groups. Moreover, we found that LV internal diameter during diastole (Figure 3D) and end diastolic volume (Figure 3E) were increased in free-fed *ob/ob* mice following IH exposure, resulting in significantly higher post-IH values compared to the other groups. These data suggest that free-fed *ob/ob* mice undergo compensatory chamber dilation in response to acute IH exposure. Even though calorically-restricted *ob/ob* mice were still obese compared to WT mice (41.8 ± 0.64 vs. 26.7 ± 0.37 g, respectively), caloric restriction was sufficient in protecting against IH-induced chamber dilation. Collectively, caloric restriction protects against LV hypertrophy and IH-induced LV dilation independent of leptin signaling.

## Discussion

Roughly half of the obese population experience OSA and IH (Framnes and Arble, 2018). OSA increases risk for hypertension, heart failure, atrial fibrillation, and other cardiovascular diseases (Drager et al., 2017). OSA is associated with elevated circulating leptin independent of body fat (Ip et al., 2000). However, obesity’s contribution to IH-induced cardiac dysfunction, independent of leptin signaling, remains unclear. Here, we exposed WT, free-fed *ob/ob*, and calorically-restricted *ob/ob* mice to 6 days of IH to determine whether acute IH exacerbates cardiac dysfunction in obese mice and whether caloric restriction can mitigate IH-induced cardiac remodeling independent of leptin signaling.

Similar to previous findings (Barouch et al., 2003), we found that free-fed *ob/ob* mice exhibited LV hypertrophy prior to IH as indicated by an increase in LV mass and posterior wall thickness. After acute IH exposure, free-fed *ob/ob* mice exhibited an increased end diastolic volume and LV internal diameter, indicating LV chamber dilation. These findings, along with a preserved ejection fraction, suggest that free-fed *ob/ob* mice exposed to IH do not exhibit systolic failure, but may be presenting with early diastolic compensation. The observed increase in end diastolic volume, stroke volume, and cardiac output in free-fed *ob/ob* mice exposed to IH suggests increased venous return to compensate for the larger oxygen demand. Increased venous return following IH can be attributed to various factors such as sympathetic increases in venoconstriction and blood volume (Izzo and Taylor, 1999), and/or an increase in baroreceptor sensitivity (Fox et al., 2006). The IH-induced elevation in cardiac work is thought to be an early adaptive response to improve oxygen delivery, but eventually may contribute to IH-associated hypertension and/or heart failure (Lucking et al., 2014). Neither diastolic nor systolic failure were directly observed in the present study. However, it is plausible that long-term exposure to IH would eventually lead to a decrease in end diastolic volume or ejection fraction, indicative of diastolic and systolic failure, respectively. Wild-type mice exposed to 8 weeks of IH, for example, exhibit LV dilation comparable to 6 days of acute IH provided to *ob/ob* mice (Chen et al., 2010). However, the longer IH exposure in wild-type mice is found to additionally decrease ejection fraction.

The observed LV chamber dilation following acute IH exposure in free-fed *ob/ob* mice is consistent with eccentric hypertrophy and may be accommodating for the LV concentric hypertrophy present prior to IH. With eccentric hypertrophy, myocyte sarcomeres stretch, and their force of contraction increases to maintain the same or higher cardiac output *via* the Frank Starling mechanism (Kemp and Conte, 2012). These findings are in line with a prior study demonstrating that 8 weeks of IH causes a switch to the eccentric signaling pathway in the myocardial tissue of rats (Chen et al., 2007).

Akin to previous findings (Xu et al., 2016; An et al., 2020), we found that 4 weeks of caloric restriction was sufficient to prevent LV hypertrophy and cardiac remodeling independent of leptin signaling. Caloric restriction protects cardiomyocytes *via* changes in cell signaling independent of weight loss (Ahmet et al., 2011). Our results agree with this notion, as calorically-restricted *ob/ob* mice were protected from cardiac remodeling despite being substantially heavier than wild-type mice. Additionally, caloric restriction may be preventing LV hypertrophy by alleviating the risk of hypertension, the leading cause of LV hypertrophy. Caloric restriction can reduce the risk of hypertension and atherosclerosis by maintaining appropriate cholesterol levels and lowering inflammatory mediators (Stein and Matter, 2011; Nicoll and Henein, 2018). One limitation of this study is that we did not use telemetry or doppler echocardiography to examine arterial or LV pressure in our animals. However, we found that calorically-restricting the *ob/ob* mice prior to IH led to a reduction in cardiac output. Since the *ob/ob* mouse is known to be hypertensive (Rodriguez et al., 2014), a reduction in cardiac output pre-IH may have decreased their arterial pressure (Magder, 2018). A reduced arterial pressure, in turn, could help prevent the development of LV hypertrophy.

While leptin was not required to prevent LV hypertrophy and IH-induced cardiac remodeling, it is possible that the presence of leptin could have been further beneficial. For example, leptin treatment, but not caloric restriction, can improve myocardial metabolism and prevent cardiac lipotoxicity in leptin-deficient mice (Sloan et al., 2011). Nonetheless, caloric restriction presents as an effective preventative measure for LV hypertrophy and acute IH-induced LV remodeling independent of impaired leptin signaling.

Our results indicate that free-fed *ob/ob* mice exposed to acute IH exhibit cardiac changes present with OSA, namely an increase in LV mass, interventricular septum thickness, and LV dilation (Yu et al., 2020; Deng et al., 2021). OSA increases one’s risk for both concentric and eccentric cardiac hypertrophy (Cuspidi et al., 2020). In the present study, we observe evidence of both concentric and eccentric cardiac hypertrophy in free-fed *ob/ob* mice exposed to IH. Moreover, in the present study, 6 days of IH exposure had no effect on ejection fraction. While a decrease in ejection fraction is observed in many patients with severe OSA, patients with mild to moderate OSA have an increased likelihood of maintaining a normal ejection fraction (Yang et al., 2012; Yu et al., 2020; Sanderson et al., 2021). Therefore, free-fed *ob/ob* mice exposed to acute IH may translate to a cardiac model of mild to moderate OSA in humans.

Our findings may be of clinical interest to the treatment of OSA, where leptin resistance is a presumed pathological feature (Framnes and Arble, 2018). Caloric restriction is known to reduce LV hypertrophy and improve diastolic function in obese individuals (Iacobellis et al., 2008). However, no studies to date have investigated the effects of caloric restriction on cardiac function in individuals with OSA. In light of the present data, future studies examining the effectiveness of caloric restriction in mitigating cardiac remodeling in individuals with OSA are warranted.

## Conclusion

Here, we find that obese, leptin-deficient *ob/ob* mice exposed to acute intermittent hypoxia develop cardiac changes associated with OSA such as LV hypertrophy and compensatory LV dilation. We find that 4 weeks of caloric restriction is effective at preventing these maladaptive cardiac changes independent of leptin signaling and body weight. Collectively, these findings may have clinical implications for OSA.

## Data Availability

The original contributions presented in the study are included in the article/Supplementary Material, further inquiries can be directed to the corresponding author.
