# Supplementary material for: Caloric restriction prevents obesity- and intermittent hypoxia-induced cardiac remodeling in leptin-deficient ob/ob mice
Source: Front Physiol. 2022 Sep 8;13:963762. doi: 10.3389/fphys.2022.963762 (PMC9493268; doi:10.3389/fphys.2022.963762)
Supplement: Supplementary file 1 [file Table1.DOCX]

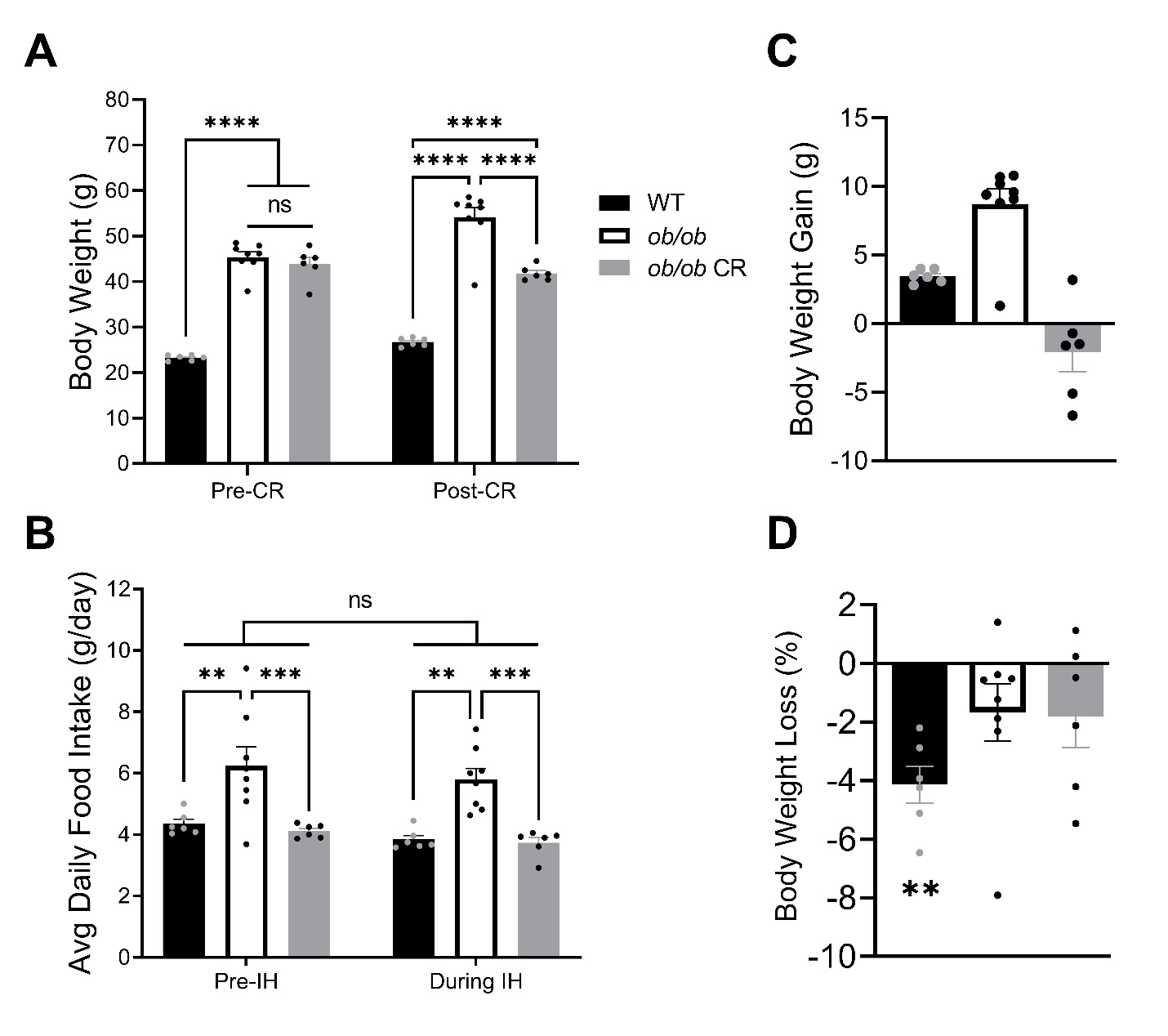
**Supplemental Figure 1A. Caloric restriction prevents weight gain in *ob/ob* mice.** (**A**) All *ob/ob* mice weighed the same prior to CR, but free-fed *ob/ob* mice (n = 8) weighed more than calorically-restricted *ob/ob* mice (n = 6) after 4 weeks of CR. (**B**) Free-fed *ob/ob* mice had a significantly higher average daily food intake compared to WT (n = 6) and calorically-restricted *ob/ob* mice during both the 6 days prior to IH and the 6 days during IH. All mice had the same average daily food intake during IH as compared to pre-IH. (**C**) Calorically-restricted *ob/ob* mice did not gain weight after 4 weeks of CR, while WT and free-fed *ob/ob* mice gained weight. (**D**) WT mice lost weight after 6 days of IH, while free-fed and calorically-restricted *ob/ob* mice did not lose a significant amount of body weight. Abbreviations: CR, caloric restriction; WT, wild-type; IH, intermittent hypoxia.
